# Implementing buprenorphine prolonged-release injection using a health at the margins approach for transactional sex-workers

**DOI:** 10.3389/fpsyt.2023.1224376

**Published:** 2023-07-20

**Authors:** Rosalind Gittins, Joseph Tay Wee Teck, Rebecca Knowles, Nicole Clarke, Alexander Baldacchino

**Affiliations:** ^1^Via, London, United Kingdom; ^2^Forward Leeds and Humankind Charity, Durham, United Kingdom; ^3^Population and Behavioural Science Research Division, School of Medicine, St Andrews University, St Andrews, United Kingdom

**Keywords:** outreach, sex-work, trauma-informed, buprenorphine, micro-induction

## Abstract

**Background:**

Access to prescribed interventions and retention in treatment services are associated with improved health outcomes and reduced premature mortality rates for people living with opioid use disorder (OUD). In Leeds, transactional sex-workers frequently cycled in and out of treatment for OUD such that they never reached a level of engagement that permitted opportunities to meet their healthcare or housing needs. Barriers to accessing care provision include an itinerant lifestyle, difficulties with travel at unpredictable hours, impacting upon adherence to medication regimens including daily supervised consumption.

**Objectives:**

To use a co-produced, “health at the margins” approach, to reach the sex-working population in Leeds, and support informed choices about the potential to receive buprenorphine prolonged-release injection (BPRI) as a treatment option for OUD.

**Methods:**

BPRI was introduced using a theory of change model and improvements in sex-worker care delivery was reviewed. Strategies included buprenorphine micro-induction, shared decision-making, collaborative multi-agency working and supporting a strengths-based and trauma-informed approach.

**Results:**

Benefits of BPRI included removal of the need for daily pharmacy visits, reducing the risk of diversion, improved medication adherence, stability and engagement with treatment and supportive services.

**Conclusion:**

BPRI may offer an additional option for pharmacological interventions for people with OUD where there may be increased barriers to accessing treatment for example due to sex-working. Strategies for effective BPRI include micro-induction, shared decision-making, collaborative multi-agency working and supporting a strengths-based approach.

## Introduction

Leeds is the fourth largest urban and metropolitan area in the United Kingdom, with an ethnically diverse population (1). Locally, there has been an increase in vulnerable people involved in street-based activities, including sex-working, and rough sleeping (2). The city has strong collaborative multi-stakeholder networks with a history of supporting creative approaches to promoting health-equity (3).

Sex-workers are an inclusion health group, which are defined as the most vulnerable and marginalized people in a community, who experience severe health and social inequities (4). Furthermore, sex-workers suffer extremely high mortality rates (with substance use as a major influencing factor) (5–8) while also being the least well investigated inclusion health group (8).

Earlier work on a managed area for street sex-work in Leeds (3) allowed an understanding of the barriers encountered by sex-workers with opioid use disorder (OUD) in accessing treatment. These barriers included an itinerant lifestyle associated with street work, difficulties with travel costs, rigid appointment times and trust in healthcare providers or meeting new professionals (9). Where treatment had commenced, attrition was often related to renewed criminal activity and imprisonment, difficulties attending the same pharmacy daily, storing medication securely, delays or interruptions in reaching an optimum dose, and other competing priorities such as alleviating withdrawal symptoms, continued sex-working, housing, access to benefits, or safeguarding issues (10–13).

Unfortunately, untreated OUD contributes to overdose deaths, sequelae from intravenous drug use including the transmission of blood borne viruses (8, 14, 15). It also reinforces the significant social harms many sex-workers already encounter such as family disruption, criminal justice involvement, homelessness and the loss of opportunities to fully participate in society (8, 14, 15).

Approximately half of people with OUD do not achieve the treatment outcomes they desire and struggle to engage with the treatment system (16). Street-based sex-workers with OUD have poorer outcomes from a broad range of treatment modalities (8, 11, 12) and drop out of treatment more frequently (10). This is due to multiple intersecting disadvantages, such as violence, homelessness, stigma, and criminalization (17), so inclusion health interventions designed to target marginalized groups more generally, do not fulfil the needs of sex-workers (4). Street sex-workers face multiple personal and structural barriers to engaging in treatment (13) and a recent systematic review found that the challenges experienced by the sex-working population are highly correlated with treatment attrition (18).

Barriers to treatment may include a worldview dominated by experiences of trauma, fear of violence, stigma, arrest or loss of privacy, negative perceptions or distrust of services, low personal efficacy, struggles with emotional regulation coupled with low frustration tolerance, withdrawal symptoms, and the impact of street life on priorities and sense of time (5, 8, 11, 12, 15, 17). From a structural perspective, issues may include the lack of gender specific services or trusted places of safety to be seen, opening hours which do not suit their lifestyle or needs, inflexible appointments, cost of transport, legal status, rigidity of program structure or institutional and staff stigma (8, 11).

Buprenorphine prolonged-release injection (BPRI) is licensed for OUD as a monthly or weekly injectable (19). It is an effective alternative to oral formulations, provides sustained therapeutic plasma levels of buprenorphine without the burden of daily dosing (especially where consumption is supervised), improved accessibility to and retention in treatment, reduced risk of medication being diverted and improved adherence and stability to pursue other health and social needs (20–23). However, there are concerns that injectable formulations may act more as a form of coercive control (20).

Local funding enabled up to thirteen sex-workers to receive BPRI. Implementation requires service level changes, including accessing funding to cover the increased cost and new medicines management processes to order, store and administer, with associated staff training and competency assessments. It also involves the design of new processes of engagement and information sharing to ensure that people with OUD are able to make a fully informed choice. Due to the potential for BPRI to significantly improve treatment retention and outcomes for sex-workers, it was critical for new engagement and information sharing processes to be designed to meet their specific needs.

There is limited peer-reviewed literature on how particularly marginalised groups such as sex-workers could be engaged with on their own terms when making decisions on novel interventions such as BPRI. Shared decision-making discussions with individuals had an added dimension of complexity, as some individuals may have struggled to access BPRI due to issues with induction onto buprenorphine, particularly if already on methadone or anxiety about experiencing withdrawals (20).

The objectives of this work were to use a co-produced, “health at the margins” approach, to reach the sex-working population in Leeds, and support informed choices about the potential to receive BPRI as a treatment option for OUD.

## Methods

### Identification of stakeholders

This work was conducted within the “Forward Leeds” specialist substance misuse treatment service, which is led by Humankind, one of the UK’s largest third-sector providers. The service has a zero threshold to entry, four hubs covering the city, including outreach teams, and strong connections with a stakeholder network supporting sex-workers. This includes Leeds City Council, West Yorkshire Police, public health, inclusion health services, specialist sex-worker addiction leads, third-sector organizations such as the Joanna Project and Basis Yorkshire. Additionally, “bridging participants” were identified: these are frontline community workers and volunteers who work closely with sex-workers, building trusted relationships within trusted spaces (24, 25). This network identified the potential benefits, barriers and facilitators in offering BPRI to sex-workers. They functioned as advocates and participated in the development, implementation and continuous refinement of the intervention.

Data on the frequency of medication for OUD re-initiation for sex-workers known to the service was used as a proxy measure to estimate numbers who may benefit from BPRI. Out of an estimated eighty-one treatment seeking sex-workers known to be accessing the service for this period, thirteen individuals required at least two re-initiation appointments and a review of clinical records indicated that they may have benefitted from BPRI. This number formed the basis of the application to commissioners for specific funding to safeguard the affordability of BPRI throughout the individual’s full treatment journey when “standard treatment” had not been effective for them and if they wished to try BPRI: otherwise, it was unethical to initiate treatment and then cease shortly afterwards due to funding.

**Figure 1:**
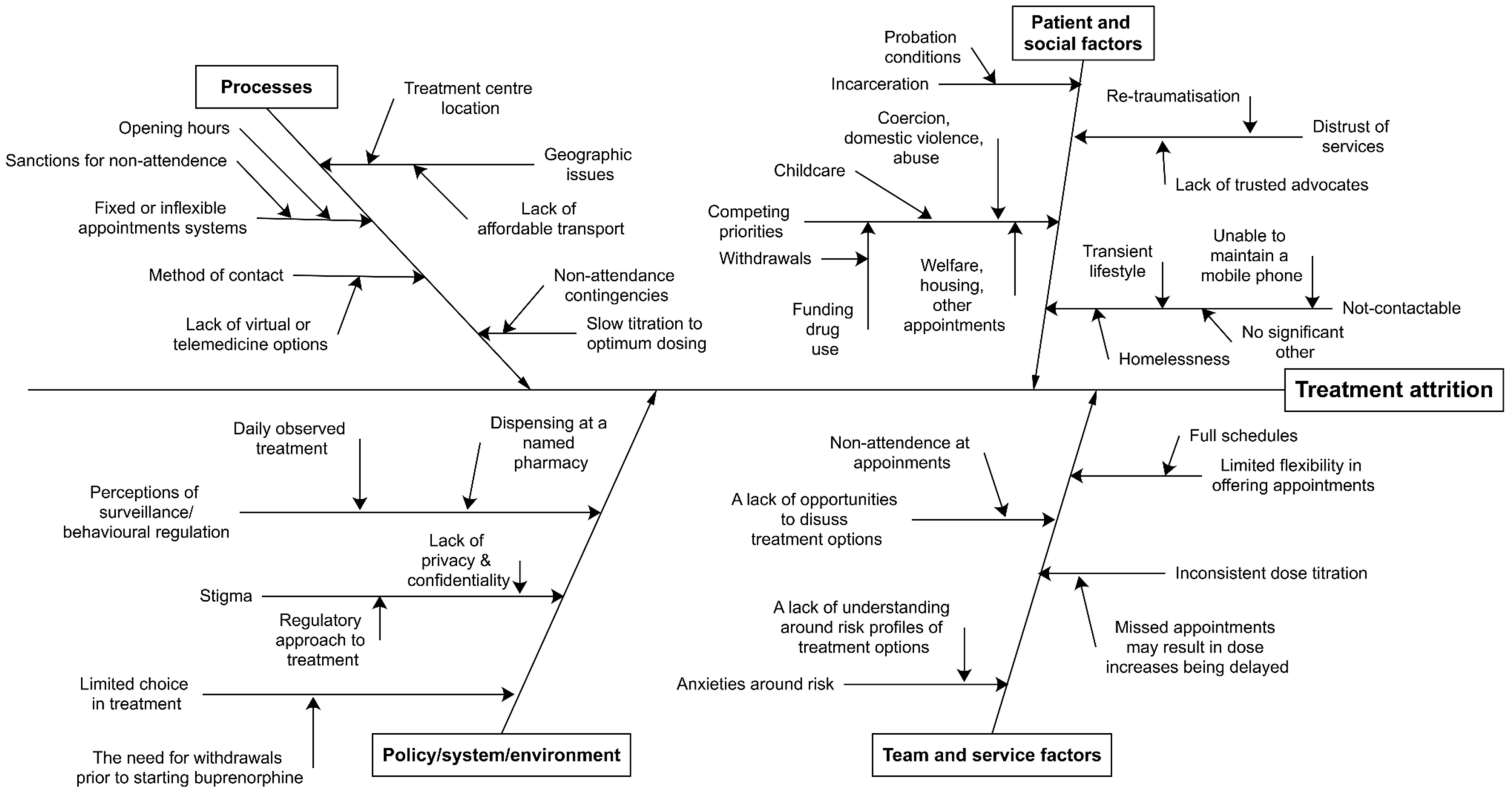
Ishikawa diagram depicting reasons for treatment attrition for sex workers accessing medication for opioid use disorder (OUD).

**Figure 2:**
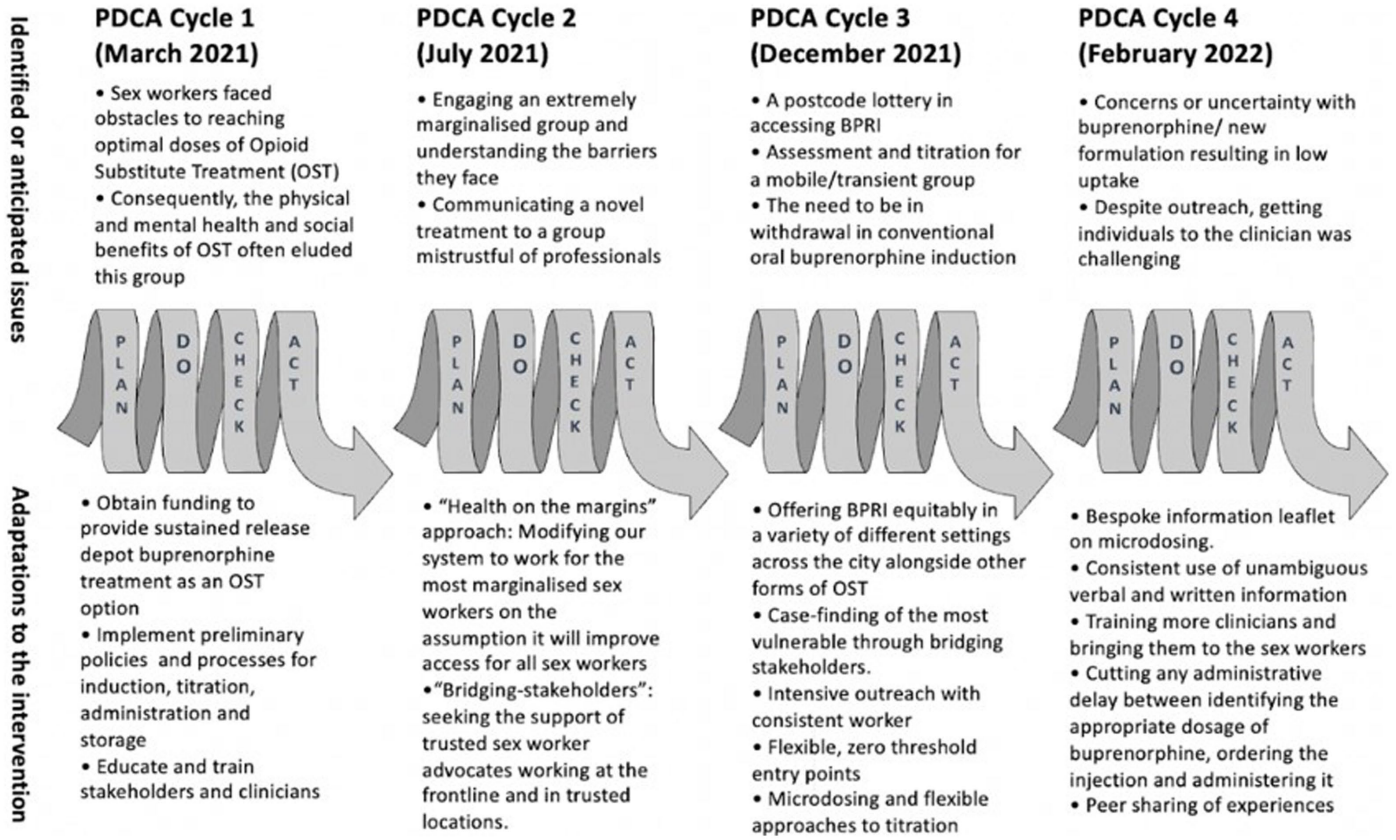
Four plan-do-check-act cycles as part of continuous improvement.

Discussions with stakeholders were processed and mapped using an Ishikawa Diagram (26) (see Figure 1). Based on stakeholder consensus, factors relating to sex-worker’s personal contexts, treatment processes, service provider characteristics, and system limitations contributing to treatment attrition were identified. An established project planning tool within health systems service improvement environments, the plan-do-check-act (PDCA) cycle (see Figure 2) (27) was then applied. This four-step model for implementing change is repeated sequentially for continuous improvement. A “health at the margins” approach was used, where the interventions were designed for the most marginalized, such that a wider implementation would be broadly inclusive of other marginalized groups (28).

### Intervention structure

**Figure 3:**
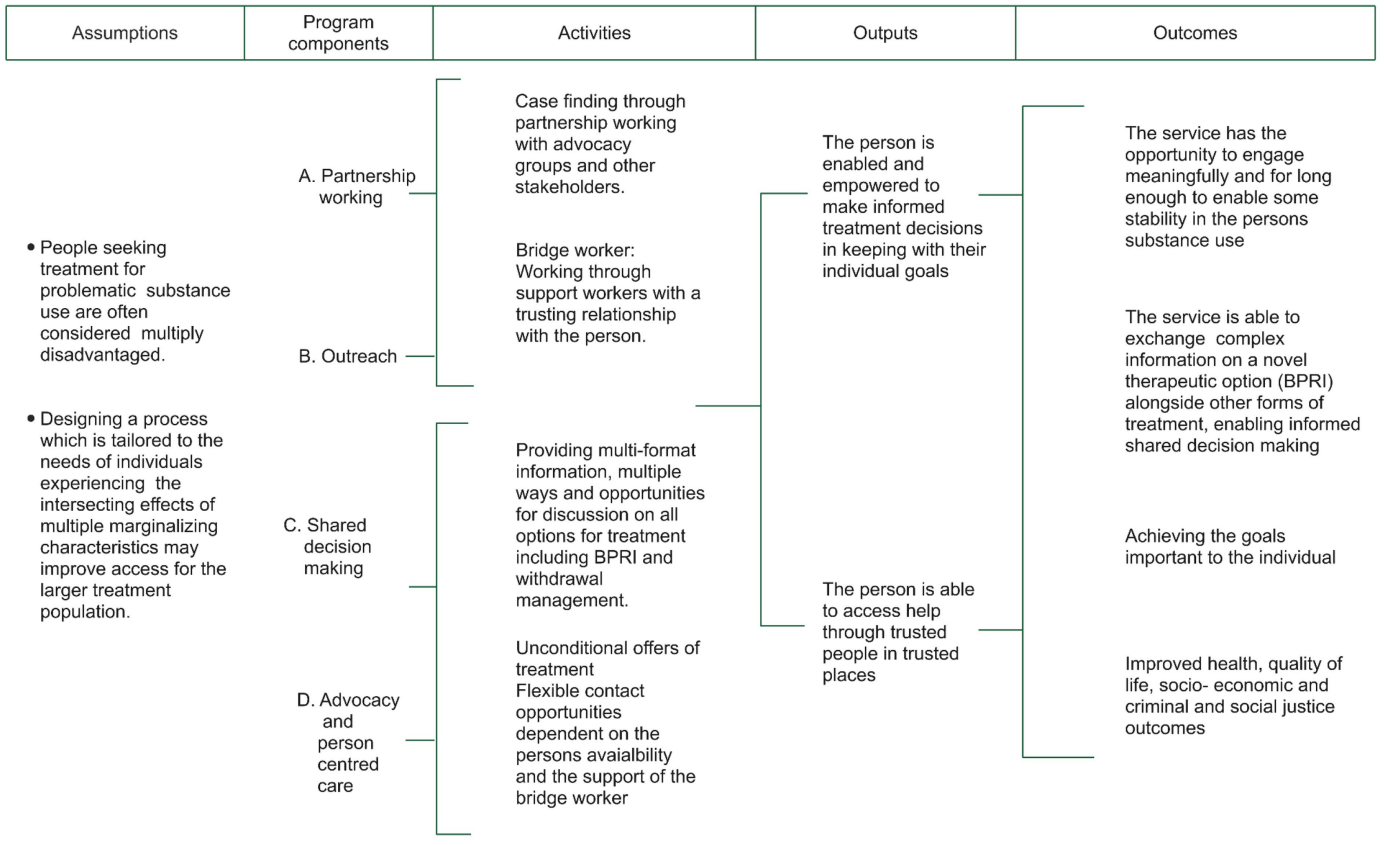
Theory of change.

There were four program components: (A) partnership working; (B) improving access through outreach; (C) choice and empowerment; and (D) advocacy and person-centred care. The program components link with the selected theory change (see Figure 3), which provides a conceptual framework of how program components and activities link to outputs/goals. This should consider all marginalized groups, with a trauma-informed approach, acknowledging subcultural and gender contexts, safety, trustworthiness, transparency, collaboration, mutuality, empowerment, choice, and control (29).

#### Partnership working

The sex-worker stakeholder network and bridging participants were essential to challenge service preconceptions of marginalization and communicating how it feels. Due to historic service delivery, pre-existing relationships with sex-workers provided insight to develop and modify the intervention. A multi-agency case management approach facilitated information exchange, enabled shared openness in decision-making, identified needs and resources, identified outcomes, reviewed processes and feedback measures, and flexibly coordinated core activities to allow delivery of the intervention.

#### Improving access through outreach

Understanding structural constraints on the individual by matching their actions with their intent is critical. Treatment providers need to be able to meet and find ways to work with individuals regardless of where they are in their recovery journey. For example, some sex-workers find repeated consultations with a clinician helpful to discuss anxieties around withdrawals and improve understanding of BPRI. Offering fixed appointment times is unhelpful and locating individuals to restrictive clinic times is difficult.

#### Choice and empowerment

Optimal shared decision making where individuals are provided with improved knowledge and access to BPRI (alongside other alternative interventions for OUD) is essential. Individuals choose and receive pharmacological interventions which are best suited to their personal goals. However, new approaches are needed to address the pharmacological peculiarities of induction onto (long acting) buprenorphine.

#### Advocacy and person-centred care

Bridging participants are core to ensuring that the voices of sex-workers are heard, and they advocate to represent their interests fairly and to rebalance “power” to ensure coercion is avoided. A key component is to support health literacy (30) so participants can critically evaluate and use it to exert greater control over their own lives and with greater autonomy. An example of this is communicating the mechanism of action of BPRI and determining the extent of drug use as a coping mechanism.

### Buprenorphine micro-induction

Oral buprenorphine (as opposed to BPRI) is an effective alternative to methadone for many years with increased use in the United Kingdom as opioid substitute treatment following the publication of the 2007 National Institute for Health and Care Excellence (NICE) Guidance (31). Unlike methadone, initiating buprenorphine in a person who is dependent on opioids requires them to be in withdrawal. Where this has not happened, the person is likely to have an induced or precipitated withdrawal when taking their first dose (32). Therefore, a person taking short acting opioids such as heroin will need to be abstinent for at least 6 hours and for long-acting products such as methadone, at least 24 hours (32).

Additionally, if someone is already taking methadone, they usually need to reduce to at least 30 mg/day before switching to buprenorphine of any formulation. This can be challenging and prolonged for individuals, placing them at risk of destabilization and is often intolerable (32). Indeed, there is growing evidence that precipitated withdrawals during buprenorphine induction may impact on long term treatment outcomes including retention in treatment and abstinence from illicit drugs (33, 34).

Micro-induction is an off-label method of administering minute doses of buprenorphine where the dose increases gradually while the person remains on their usual full agonist opioid, avoiding the need for a washout period (32). The method can be complicated and requires careful communication with the individual such that the regime is followed carefully. Nevertheless, offering this option makes BPRI accessible where buprenorphine is their preferred treatment option. The patient information leaflet used for individuals interested in the micro-induction option can be found in the Supplementary material.

### Ethical considerations

This project was considered as regular quality improvement service activities conducted within the established organizational policies and procedural frameworks, so was registered as a Quality Improvement Project with the organizations internal ethics oversight committee. It was exempt from a formal ethical review as no identifiable data was used.

## Results

Due to the small sample size, descriptive data is reported in accordance with SQUIRE 2.0 guidelines (35). Table 1 describes the findings in detail. Barriers and facilitators identified in this project were mapped against the consolidated framework for implementation research (CFIR) (36).

**Table 1:** Barriers and facilitators to implementation classified according to consolidated framework for implementation research constructs.

Construct	Barriers	Facilitators
*I. Intervention characteristics*		
Intervention source	Challenges with identifying needs in this highly vulnerable group.	The intervention was designed in response to identified frontline and advocacy worker gaps in service for sex workers.
Evidence strength & quality	Limited peer reviewed literature on the use of buprenorphine prolonged-release injection (BPRI) in the sex worker population.	Substantial evidence and experience of outreach approaches for engaging sex workers.
Adaptability	As a controlled Drug (CD), the chain of custody of the medication had to be maintained and CD regulations adhered to. The dosing schedule of the medical product is defined in its product licence.	The outreach and buprenorphine induction elements were revised in a cycle of continuous improvement. The dosing regimens for weekly/monthly injections have a window of administration before/after the due date. Humankind has established policies which support off-label use and outline CD, prescribing and administration processes.
Trialability	Staff workload pressures and clinical priorities can limit capacity to trial new approaches.	The project was designed on the basis of multiple PDCA cycles of improvement.
Complexity	The assessment and induction onto buprenorphine could be complex at times, particularly if micro-induction was required. Both frontline workers and sex workers found the regimes difficult to understand.	Once the correct dose of buprenorphine was confirmed, the dosing regime for the BPRI were easily understood and applied. Bespoke micro-induction information leaflets were co-produced.
Cost	The BPRI was notably more expensive than other conventional opioid substitute treatment. As the injections were ordered on a ‘named patient’ basis, any missed doses could entail significant wastage costs.	Our commissioners were supportive of the project and committed funding to support up to 13 sex workers on the medication.
*II. Outer setting*		
Individuals needs & resources	Resource and capacity issues may limit the ability to meet essential needs for example housing.	The managed approach in Leeds and the experiences of the bridging stakeholders provided a good understanding of the needs of the sex workers.
Cosmopolitanism	Acknowledgement of people requiring an individualised approach.	The service has proven effective collaborative relationships with agencies providing care, support and advocacy to sex workers.
External policy & incentives	At present there is limited budget to afford this intervention for wider implementation.	A new drug strategy has been introduced across England with a substantial funding drive towards offering innovative treatment options. Recent cost-benefit analysis supports BPRI as a cost-effective option.
*III. Inner setting*		
Available resources	Ongoing recruitment and staff turnover restricted training opportunities at times.	Our organisation had experience in implementing BPRI in other settings and so had training materials, information leaflets and policy documents in place.
Access to knowledge & information	Initial materials such as information on micro-induction was lacking.	Bespoke information leaflet produced on micro-induction with a co-production approach.
*IV. Characteristics of individuals*		
Knowledge & beliefs about the intervention	Knowledge is primarily theoretical and Leeds specific implementation experience was limited.	Experience gained by absorbing learning from successful implementations elsewhere, including from staff who have worked elsewhere.
*V. Process*		
Planning	Resource and capacity issues may limit the ability to attend meetings, further compounded by the COVID-19 pandemic.	Considerable pre-implementation planning took place including confirmation of funding, training and supply chain arrangements.
Champions	Acknowledgement that designated engagement roles may be required.	Each service location had a sex worker lead who worked closely with potential participants and third sector partners.

### PDCA cycles

PDCA cycles were used to test and refine the interventions. Regular stakeholder meetings informed each cycle in terms of reviewing progress and discuss next action steps. All PDCA testing occurred from March 2021 to February 2022.

#### PDCA cycle 1

Considerable time was spent in disseminating BPRI information, addressing misconceptions and exploring with stakeholders what barriers needed to be overcome. This work was undertaken during the COVID-19 pandemic which impacted staffing and operational adaptations to allow social distancing and infection control measures. Despite this challenging context this innovation was pursued as sex-workers were at a substantially greater risk of direct and indirect pandemic-related harms (37, 38).

#### PDCA cycle 2

The biggest challenge was to establish trust, communicating complex information, and working with “unconventional goals” (39, 40). Lack of understanding about BPRI amongst people who use drugs was noted (41). Specifically, sex-workers may need to balance the potential freedom BPRI gives them against their self-determination regarding when and how to use drugs and what effect is desirable to them. Furthermore, services need to be diligent to avoid coercion where the use of BPRI is found to be of greater interest or benefit to providers or the pharmaceutical company rather than the individual.

#### PDCA cycle 3

People who are extremely marginalized are also often easily ignored (42, 43). It was essential therefore that all possible measures to reach the target population were explored. This was achieved by working closely with bridging participants, active case-finding and management, intensive outreach, zero or low threshold entry into services and minimizing any delays in induction.

#### PDCA cycle 4

Bridging participants provided sex-worker perspectives, disseminated information on the intervention, helped identify the most marginalized and easily ignored individuals, and supported attendances at appointments. As many sex-workers experienced a combination of intoxication, homelessness, hunger, poor health, complex lives, and difficulty focussing on anything other than short-term survival, particular care was required to ensure that participants were able to make an informed and uncoerced decision regarding BPRI. Bridging participants ensured information was understood and facilitated an open dialogue due to their years developing trusting relationships with known sex-workers.

### Measures

Initial measures included the number of sex-workers reached and informed on BPRI as an option and the number who decided to accept the intervention. From this, more detailed comparative data focussed on individuals who initiated BPRI and their progress. These data included outcomes such as dose optimization on preferred OUD medication, interruptions to treatment, retention in treatment, abstinence from substance use and attainment of personal treatment goals both pre and post intervention. The need for novel treatment initiation via micro-induction using oral buprenorphine, their satisfaction with this, and BPRI administration was examined.

Forty-three sex-workers were offered BPRI. Twelve declined, citing fears of the unknown and not having prior knowledge of BPRI. Some felt that BPRI was “too new,” as none of their immediate peers had experienced it. Six women have now made the transition to BPRI, and as they share their experiences, more of the target population may want this intervention. An issue which delayed progress was bringing the needed expertise (for example a clinician) to the sex-worker at a trusted location.

### Individuals’ experiences

For the six individuals, getting to this point in their lives seemed impossible before this intervention. All have gained weight and are engaging with healthier lifestyle choices. They have independently reported feeling cared for and “chosen” and having the opportunity to be part of the intervention gave them hope. Five are no longer sex-working and the one individual who continues only works occasionally and is engaging with specialist sex-worker and mental health support services. One individual has converted from BPRI to oral buprenorphine as she plans to move out of area and is now engaging more widely with community support. Three individuals that are accessing mental health support are no longer using heroin, though two are intermittently using other substances (alcohol and crack-cocaine), reportedly to help manage their emotions. More detailed preliminary feedback is available from two women:

Person one: a 35 years-old woman in treatment for 6 years and with a history of drug use of 16 years. She has a history of trauma and comorbid mental health issues. On transitioning from generic sublingual buprenorphine to BPRI, she became abstinent from illicit drugs, allowing the possibility of stopping sex-work. She has started volunteering and now spends money on caring for herself, for example on food.

Person two: a 38 years-old woman who started using heroin at the age of 18. As she was on 80 mg of oral generic methadone solution, she opted for micro-induction to move on to BPRI, from which she has now completed detoxification. She is now preparing to commence naltrexone for relapse prevention, stopped sex-work, working on her fitness and has booked a trip abroad to visit family.

### Discussion

This project was able to demonstrate effective partnership working to meaningfully engage sex-workers in treatment for OUD and associated health and wellbeing interventions. We have outlined a model of care delivery that has the potential to significantly change and improve upon the current sub-optimal standard of care that this vulnerable group typically experience. Our approach to the use of PDCA cycles has also allowed us to demonstrate the positive implementation of quality improvement methodology in clinical service delivery for the benefit of individuals and in keeping with the drive to implement evidence-based decision making and reduce inefficiency (44, 45).

In accordance with NICE guidance (46), this project highlighted the importance of shared decision making and ensuring that people have sufficient information to provide informed consent for prescribed interventions. This is demonstrated by the co-production of a bespoke information leaflet when sex-workers and frontline staff disclosed struggling to understand the novel concept of micro-induction. The innovative approach outlined in this work is in keeping with the latest United Kingdom government strategy: “From Harm to Hope” and remain optimistic that the allocated additional funding for specialist treatment services will facilitate further roll-out (47). While service providers may focus on the novelty of BPRI, it is essential to note that the sex-workers we have interacted with were drawn to the care that was offered to them and their understanding that the offer of this intervention was evidence of their value.

For successful implementation, staff capacity, robust communication with partner agencies, training and education of stakeholders and collaborative working in the form of a community of practice were essential. Assertive outreach approaches, the use of bridging participants, the processes underpinning the delivery of BPRI and off-label interventions such as buprenorphine micro-induction needs to be in place in advance. Barriers to implementation were compounded by the COVID-19 pandemic. Ongoing advocacy to provide equitable and accessible services to sex-workers with ring-fenced funding is required. Given the relative medication costs of BPRI and clinician staffing levels required to facilitate prescribing and administration, it is important to have a clear understanding of opportunity costs and trade-offs in pursuing this intensive intervention as opposed to oral medication for OUD.

Early, iterative minor changes led to meaningful improvements. Mapping barriers and facilitators against the CFIR (36) standardised multilevel implementation contexts that influence all stages of an intervention and increased the generalizability of our findings. We have demonstrated that frontline practice-based experience can compensate for the lack of standard peer reviewed research when carrying out quality improvement or implementation research with marginalized groups (4, 48). We relied heavily on our bridging participations and on the experiences of our third-sector partners (49) in bringing this privileged knowledge into the intervention. Critically, this was shared through a community of practice, shaped by a shared domain of knowledge, a social network of learning, common tools and frameworks, often used to disseminate evidence based best practice within the health sector (50).

Within the context of applying a trauma-informed model of care, there are two major omissions which should be rectified in upcoming PDCA cycles. The first is the absence of “survivor partnerships” (29) to co-produce this intervention and to provide peer support for the participants. The second is the provision of rapid and established pathways to trauma-specific care, should this be desired. While we did have access to mental health led trauma support, we did not have a fast track into this service, a potential issue should the participant struggle due to the absence of the effect of a full opioid agonist.

Another key issue is the greater cost of BPRI compared with long established medications such as oral methadone and buprenorphine. We were fortunate to have dedicated pharmacy input to calculate and negotiate medication costs and support with implementing models of BPRI use among the OUD treatment seeking population. It is also important that some recent favourable cost-benefit analyses (21, 51) coupled with comparable outcomes to conventional oral treatment (20) are some of the drivers for the growing national interest in BPRI. The possibility of upskilling frontline workers to support virtual consultations, with the administration of the injection being carried out by health care assistants or Pharmacy Technicians under the legal framework of a patient specific direction is being considered.

Since sex-workers experience multiple disadvantages, inequalities in access to services, and poor health, social, economic, housing and criminal justice outcomes (11, 12, 17, 38, 42, 49) our work highlights the need for a reviewed approach to the provision of interventions for this group, especially for street sex-workers with OUD, who are known to carry the greatest burden of morbidity and mortality (7, 11, 12). We plan to share further outcomes of this work as the service delivery model becomes further embedded and refined, and cascaded across other service delivery sites, shared with commissioners and other specialist treatment providers. We also acknowledge the current paucity of the published evidence base relating to off-label micro-induction, (especially with consequent BPRI induction) and further research which considers the use of this pharmacological intervention in this way requires further exploration. Currently we are aware of pockets of innovation which mirror this piece of work (32).

## Conclusion

This project is an exemplar of the use of quality improvement frameworks such as PDCA cycles and the application of the CFIR to improve clinical service delivery. Furthermore, how BPRI (including micro-induction) can be utilized effectively in the complex sex-worker cohort is described. Finally, and arguably the most critical finding from this project, is that innovation and new technology play a secondary role to person-centred, trauma-informed care, shared decision making and close partnership working with stakeholders when engaging extremely marginalized groups such as sex-workers.

## Data availability statement

The original contributions presented in the study are included in the article/Supplementary material, further inquiries can be directed to the corresponding author.

## Ethics statement

Ethical review and approval was not required for the study on human participants in accordance with the local legislation and institutional requirements. The ethics committee waived the requirement of written informed consent for participation.

## Author contributions

JT, RG, and RK were responsible for the design and implementation of the project. JT provided direct clinical care, supervision, and training. RG provided training, clinical governance and pharmacy oversight when employed by Humankind. RK led, coordinated, and managed the multi-agency joint working and outreach aspects. NC and AB provided advice and feedback on developing the manuscript. All authors contributed to the article and approved the submitted version.

## Conflict of interest

The authors declare that the research was conducted in the absence of any commercial or financial relationships that could be construed as a potential conflict of interest.

## Publisher’s note

All claims expressed in this article are solely those of the authors and do not necessarily represent those of their affiliated organizations, or those of the publisher, the editors and the reviewers. Any product that may be evaluated in this article, or claim that may be made by its manufacturer, is not guaranteed or endorsed by the publisher.
