# Supplementary material for: Implementing buprenorphine prolonged-release injection using a health at the margins approach for transactional sex-workers
Source: Front Psychiatry. 2023 Jul 20;14:1224376. doi: 10.3389/fpsyt.2023.1224376 (PMC10400437; doi:10.3389/fpsyt.2023.1224376)

**Supplementary Information**

Micro-induction Patient Information Leaflet


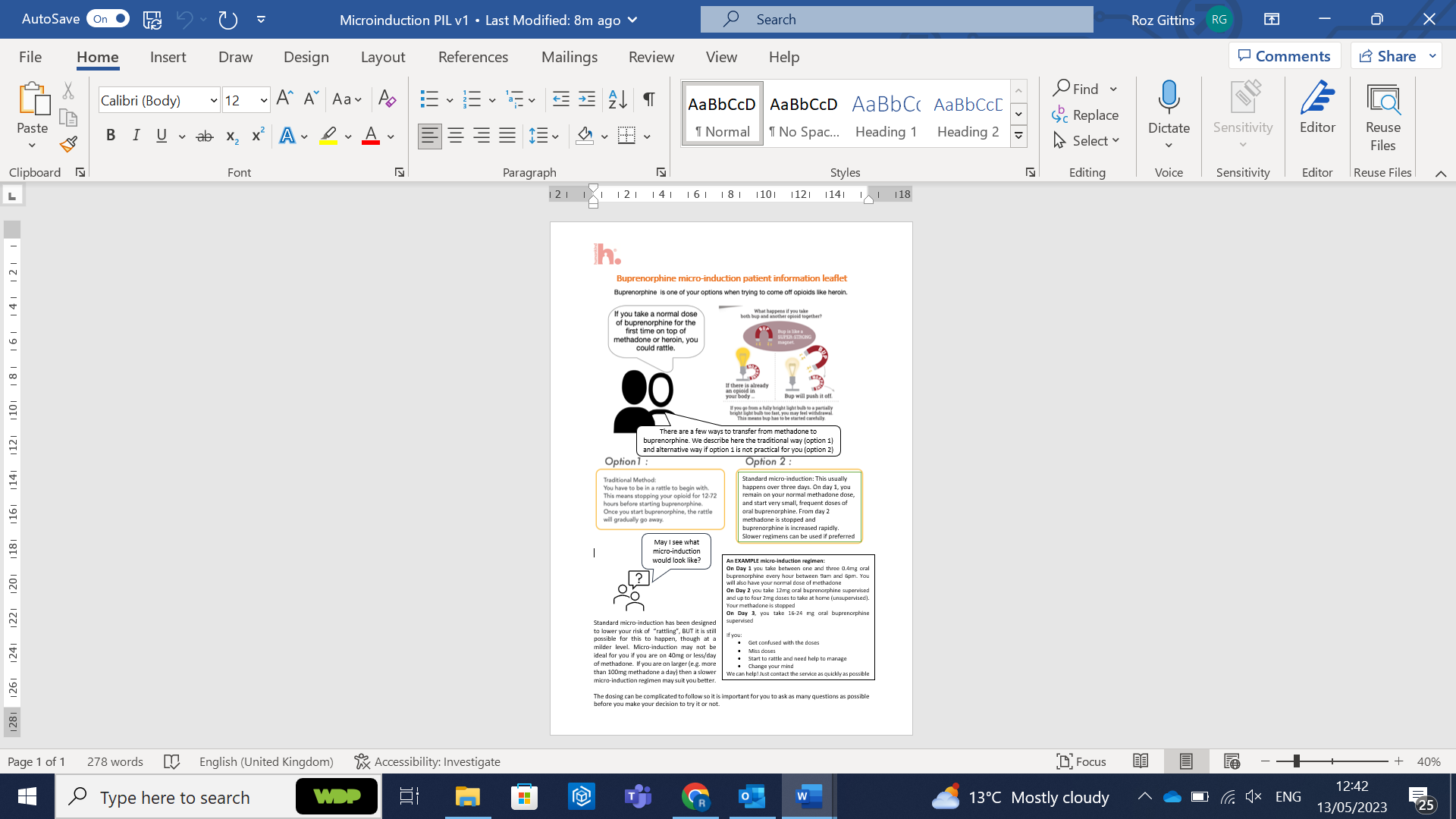


Micro-induction Dosing Schedules


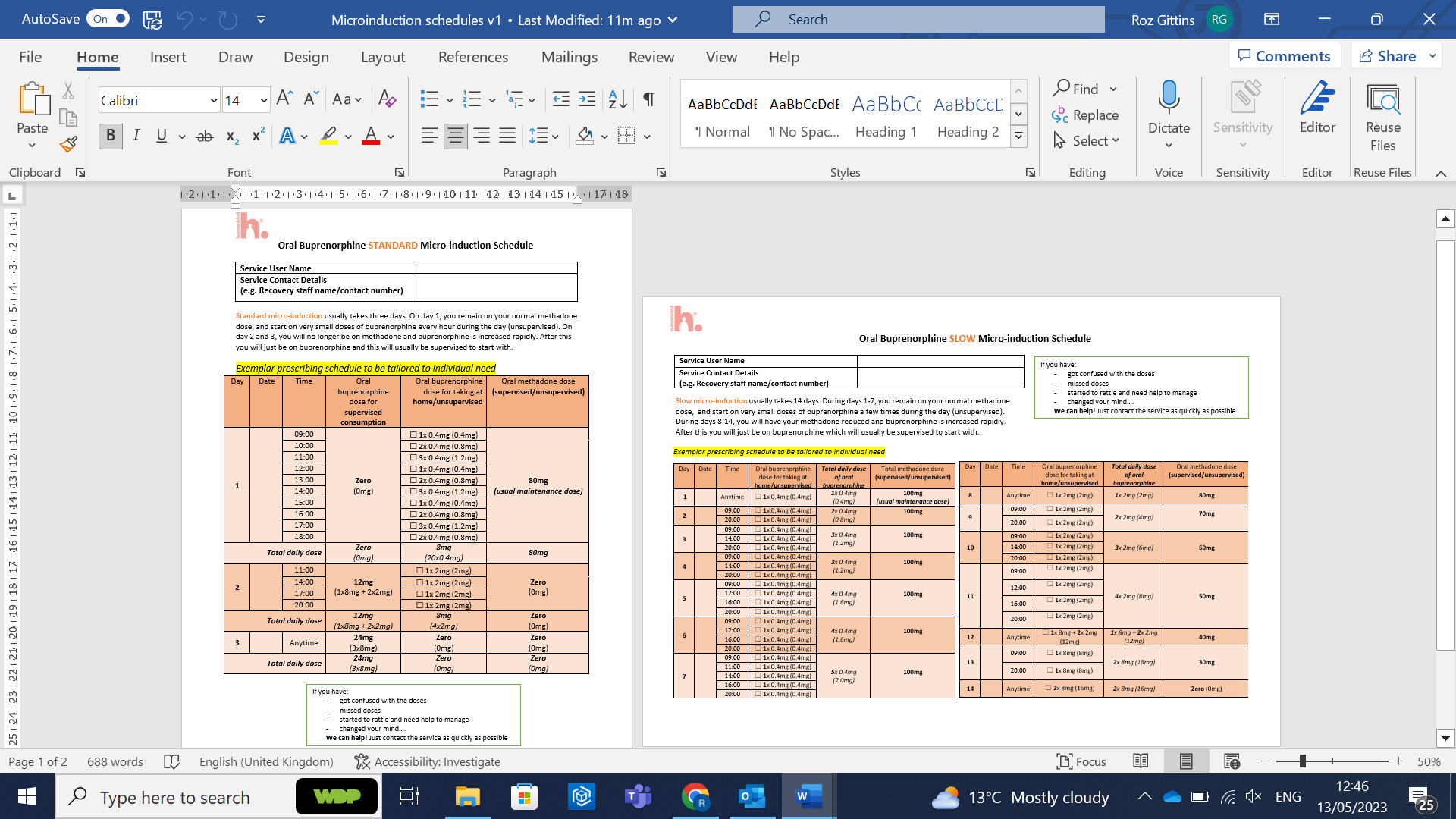


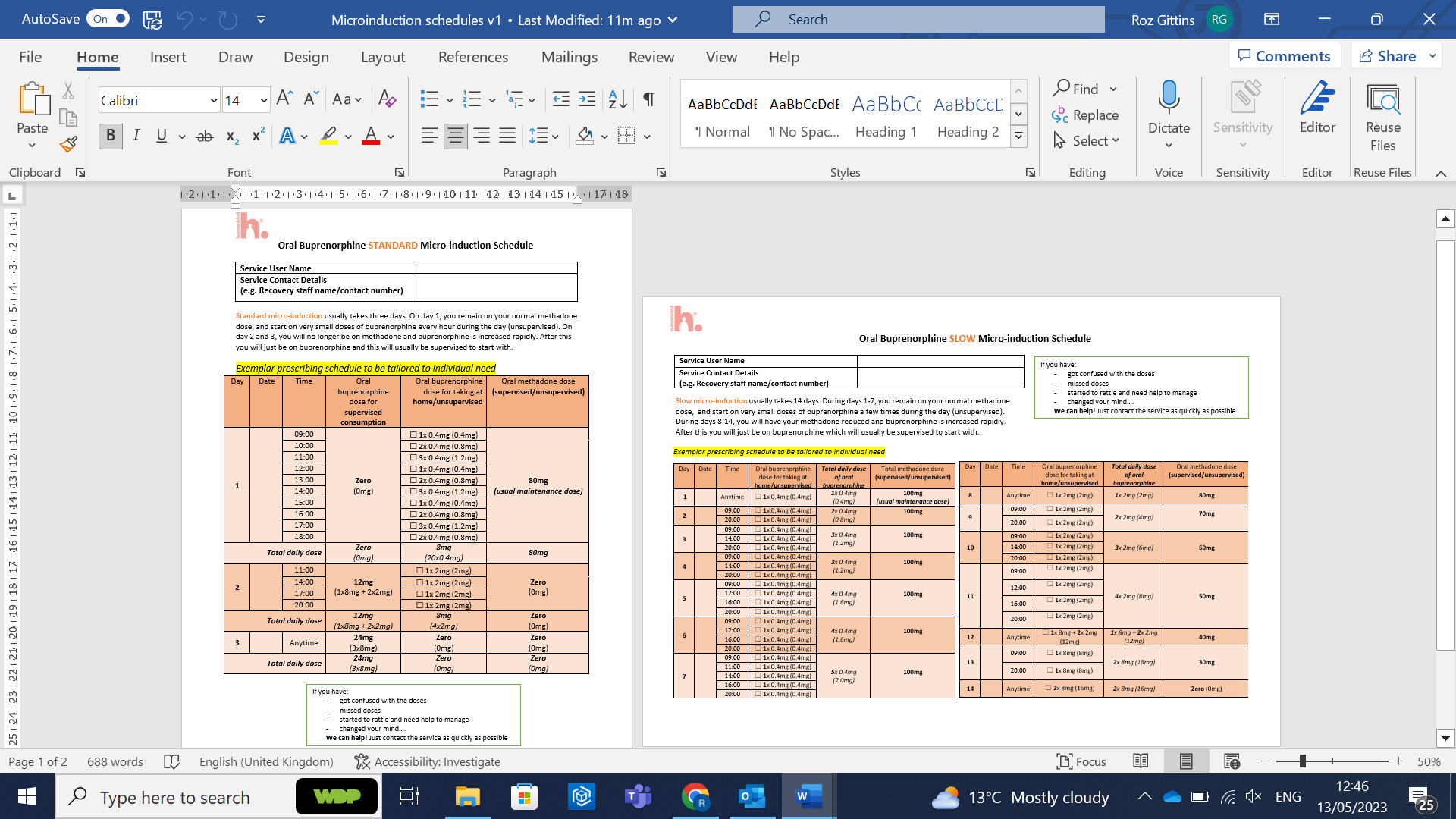

Supplement: Supplementary file 1 [file Data_Sheet_1.DOCX]
